# Functionalized Mouth‐Conformable Interfaces for pH Evaluation of the Oral Cavity

**DOI:** 10.1002/advs.202003416

**Published:** 2021-03-18

**Authors:** Giusy Matzeu, Gili R. S. Naveh, Siddhart Agarwal, Jeffery A. Roshko, Nicholas A. Ostrovsky‐Snider, Bradley S. Napier, Fiorenzo G. Omenetto

**Affiliations:** ^1^ Silklab Department of Biomedical Engineering Tufts University Medford MA 02155 USA; ^2^ Center for Applied Brain and Cognitive Science Tufts University Medford MA 02155 USA; ^3^ Laboratory for Living Devices Tufts University Medford MA 02155 USA; ^4^ Harvard School of Dental Medicine 188 Longwood Avenue Boston MA 02115 USA; ^5^ Department of Electrical and Computer Engineering Tufts University Medford MA 02155 USA; ^6^ Department of Physics Tufts University Medford MA 02155 USA

**Keywords:** colorimetric sensors, mouth compatible interfaces, oral cavity

## Abstract

Oral health monitoring is highly desired, especially for in home use, however, current methods are not sensitive enough and technically convoluted for this purpose. This paper presents incorporation of bioactive materials and colorimetric chemical sensors into routinely used oral appliances transforming them into bioresponsive, conformable interfaces. Specifically, endodontic paper points and dental floss can be functionalized to locally sense and monitor pH variations within the oral cavity via color changes. Moreover, edible colorimetric indicators are developed and used to make sensing, edible devices in the form factor of candies that can dynamically and visually respond to acidity changes in saliva. These interfaces would enable early detection of caries (e.g., using dental floss and paper points) providing low‐cost point of care devices that respond in real‐time by detecting pH variations in biological fluids thus bringing monitoring to home settings .

## Graphical abstract

pH‐responsive biomaterial‐based formulations based on fruit can be tuned to convert inert interfaces such as dental floss and highly absorbent paper points into devices for real‐time localized chemical mapping of the oral cavity. These can be extended to mouth‐conformable interfaces to acquire information on the health status of the user and for the design of point of care diagnostic devices.

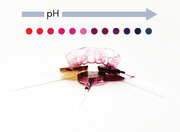

The oral cavity provides an exceptional diagnostic environment. It is readily accessible with minimal to no invasiveness, offering access to blood as well as to mucosal samples from the tongue, cheeks, and pharynx.^[^ ^1^ ^]^ Moreover, the oral cavity allows the collection of saliva with proficiency and minimal efforts.^[^ ^2^ ^]^ The noninvasive nature of saliva detection in real‐time makes oral biomarker evaluation a potentially inexpensive and easy to use diagnostic approach.^[^ ^3^ ^]^ In the clinical practice, saliva evaluation currently allows one‐time measurements thus preventing continuous multianalyte tracking and, in many instances, causing diagnostically indicative fluctuations in local parameters to be missed. Despite the great potential, salivary diagnostics for domestic use has not yet transitioned into commercially available devices. The main limitations in salivary diagnostics are dictated by the lack of reproducible sampling techniques,^[^ ^4^ ^]^ analytical computation of low concentrations (i.e., range of pg–ng per µL) and dynamic levels of biomarkers intra‐ and interindividuals.^[^ ^5^ ^]^ Moreover, biomarkers levels and saliva composition vary throughout the day (e.g., following hormonal oscillations) making it an excellent tool for personalized diagnostics on one hand, however, establishing the diagnostic correlations between analyte variations and specific diseases limited on the other.^[^ ^6^ ^]^

Saliva is a hypotonic exocrine fluid consisting of 99% of water and is the initiator of the digestion process. It mainly contains electrolytes, proteins, immunoglobulins, viral and bacterial genetic codes, as well as antimicrobial regulators and lubricating agents.^[^ ^7^ ^]^ Saliva is also an ion reservoir for oral pH regulation and enamel remineralization (i.e., maintaining neutral pH levels within the range 6.6–7.1).^[^ ^8^ ^]^ pH fluctuations can compromise oral health and specifically the tooth structure leading to the most prevalent oral disease: dental caries,^[^ ^9^ ^]^ a multifactorial transmittable infectious pathology resulting from an imbalance between acidogenic bacteria, dietary intake, and salivary remineralization capacity. A continuous monitoring of the pH levels in specific locations will be the most indicative for increased demineralization process. Provided with adequate concentration of calcium, phosphate, and fluoride ions, enamel demineralization can be reversed.^[^ ^10^ ^]^ Salivary pH below 6.6 is indicative of increased risk for dental caries:^[^ ^11^ ^]^ localized monitoring, which targets increase in acidic levels (i.e., below pH 5.5) between and around teeth can be exploited to indicate either the onset or presence of caries.^[^ ^11^, ^12^ ^]^

Current detection methods such as clinical examination and X‐rays can identify established carious lesions and are only available in dental practices. Therefore, platforms that can controllably access specific areas of the oral cavity and dynamically monitor pH fluctuations outside of dental offices are of vast importance.^[^ ^13^ ^]^

Electrochemical sensors offer promising solutions for monitoring pH variations in saliva but are nowadays constrained to laboratory settings. However, advancements in the realization of flexible, miniaturized electronic devices may allow the access of the oral cavity and become of paramount efficacy in evaluating mouth conditions (e.g., biochemical analytes, physical variations, etc.) in the near future.^[^ ^13^, ^14^ ^]^

Colorimetric sensing,^[^ ^15^ ^]^ in spite of its known limitations, may offer a compelling strategy for practical and rapid detection of local pH variations^[^ ^16^ ^]^ inside the mouth. We present here an approach based on the use of biomaterial‐based mixtures to functionalize inert interfaces of common dental appliances such as dental floss and endodontic paper points to diagnose local areas of the oral cavity. We also demonstrate bioresponsive colorimetric candies that embed color changing biochemical reporters to continuously transduce pH variations in saliva. The sensing formulations based on naturally derived biomaterials provide the advantage of using water as a solvent and of being processed at room temperature, thus enabling direct integration and stabilization of labile sensing molecules^[^ ^17^ ^]^ into cocktails based on a liquid suspension of silk fibroin.^[^ ^18^ ^]^ Such cocktails have the capability to stabilize commercially available pH indicators or labile pH sensing molecules (e.g., anthocyanin, carotenoid, etc.) extracted from fruits (e.g., blueberries (BB), nectarine skins) and vegetables (e.g., red cabbage (RC)).^[^ ^19^ ^]^ They can be tuned for spray‐coating and dip coating or to create solid bioreactive interfaces with long shelf‐life under ordinary (i.e., refrigeration‐less) storage conditions without compromising their biochemical reporting functionality.^[^ ^18^, ^20^ ^]^

The versatility of the approach is shown in **Figure** **1**. The principle of operation is to combine bioreactive formulations (see the Supporting Information for details on formulations and viscosity) with traditional tools commonly used in dentistry by either coating or incorporation of reagents onto dental floss (i.e., through spray coating), onto highly absorbent paper points used for root canals (i.e., by dip coating), and by developing standalone reactive candies by direct inclusion of fruit‐extracted reagents.

**Figure 1:**
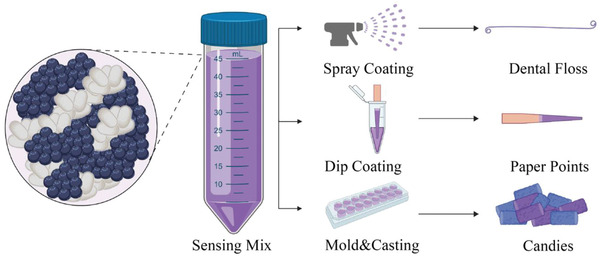
Biomaterial‐based sensing mixes consisting of silk fibroin and pH sensing molecules (i.e., commercially available pH indicators or extracted from fruits and vegetables). The schematic shows the steps involved in the making of sensing interfaces that can be used to monitor pH variations within the oral cavity. Sensing mixes contain silk fibroin and a pH sensing molecule. The sensing mix is used to realize different types of intraoral sensing devices: i) spray coated to generate pH detecting dental floss; ii) dip coated highly absorbent paper points to detect pH in between teeth or inside teeth during root canal treatment; iii) casted into molds to realize color changing candies able to sense pH variations in user's salivary fluid.

The biomaterial‐based bioresponsive formulations were first characterized in liquid format via UV–vis spectrophotometry (Figures S1–S8, Supporting Information). Analytical performance was preserved when all the pH indicators were integrated into silk fibroin‐based mixtures thus validating the possibility for their use to activate inert substrates of different kinds as described above (see the Supporting Information for additional details).

The biomaterial‐based formulations were first used to encapsulate commercially available colorimetric pH‐responsive molecules, namely, a combination of bromocresol green (BG)/chlorophenol red (CPR), and nitrazine yellow (NY), which were spray coated on dental floss (**Figure** **2**a,c,e). pH variations were monitored in real‐time by measuring variations in the intensity of red, green, or blue (RGB) channels or of a combination of the three of them, by measuring the Euclidean distance (ED) of their colorimetric response. The color channels exhibited varying levels of sensitivity (sensitivity RED: −33.8 ± 1.5, *n* = 3, NY; sensitivity RED: −26.6 ± 1.6, *n* = 3, BG/CPR). Figure 2a,e shows variations of Red intensity for both NY and BG/CPR within the pH range 6–8 and 3–6, respectively (see the Supporting Information for additional details). Figure 2c shows how different areas of the same dental floss can colorimetrically detect different pH values (i.e., pH 4, 6, and 7.5) allowing for accurate real‐time tracking of intertooth pH variations, which is particularly important to monitor given the difficulty to reach these areas with conventional oral hygiene.

**Figure 2:**
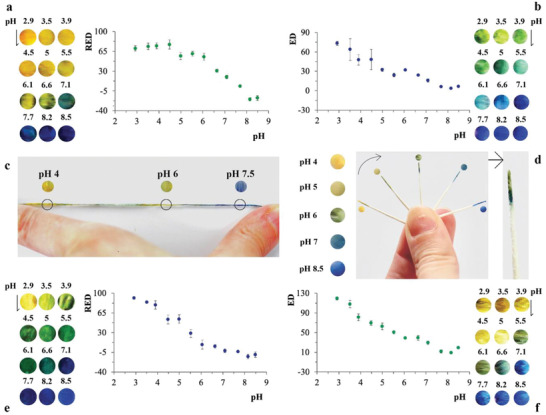
Characterization of biomaterial‐based sensing mixes used a,c,e) to spray coat dental floss and b,d,f) to dip coat highly absorbent paper points able to colorimetrically detect pH variations within the oral cavity in real time. The plots and pictures show the color changes of indicators at pH values ranging between 3 and 8.5. a) Nitrazine yellow (NY) embedded into a silk‐based mix spray coated on dental floss: sensing range pH 6–8 (*n* = 3, error bars standard errors). Colored circles show the colorimetric response recorded at different pH (indicated by the label above circular areas). b) Mix of bromocresol green (BG)/chlorophenol red (CPR) embedded into a silk‐based mix used to dip coat highly absorbent paper points: sensing range pH 3–5.5 (*n* = 3, error bars standard errors). Colored circles show the colorimetric response recorded at different pH (indicated by the label above circular areas). c) Mix of BG/CPR spray coated on dental floss. The picture shows the dental floss exposed to different pH variations in three highlighted sections: enlargements at pH 4, 6, and 7.5 (i.e., from left to right). d) Highly absorbent paper points dip coated with NY embedded into silk‐based mixes. NY color changing points were exposed to pH 4, 5, 6, 7, and 8.5 (i.e., the arrow indicates the direction of paper points exposed to increased pH). Right: Enlargement of paper point at pH 6. Colored circles show the colorimetric response recorded at different pH (indicated by the label next to circular areas). e) BG/CPR embedded into a silk‐based mix used to spray coat dental floss: sensing range pH 3–6 (*n* = 3, error bars standard errors). Colored circles show the colorimetric response recorded at different pH (indicated by the label above circular areas). f) NY embedded into a silk‐based mix used to dip coat highly absorbent paper points: sensing range pH 3–7.5 (*n* = 3, error bars standard errors). Colored circles show the colorimetric response recorded at different pH (indicated by the label above circular areas). The plots allow quantifying the signal as variations in either Red (i.e., CPR/BG and NY for dental floss) or Euclidean distance (ED) (i.e., CPR/BG and NY for paper points) channel intensity versus pH.

The same commercially available pH indicators were used to dip coat highly absorbent paper points that are currently employed for standard clinical treatment (i.e., root canal) (Figure 2b,d,f). Color changing paper points may allow localized monitoring of pH variations in hardly accessible areas inside the oral cavity and inside the teeth. Figure 2b,f shows intensity variations of ED for both BG/CPR and NY within the pH range 3–8.5. BG/CPR points are sensitive within the range pH 3–5.5 (sensitivity ED: −18.3 ± 0.4, *n* = 3) and NY points are sensitive within the range pH 3–7.5 (sensitivity ED: −20 ± 0.5, *n* = 3) (see the Supporting Information for additional details). Figure 2d shows the colorimetric response of paper points at different pH, with the insets highlighting performance at pH 6 (i.e., for NY points).

BG/CPR and NY were selected to functionalize both dental floss and paper points due to their overlapping p*K*_a_ over the range of interest for noninvasive monitoring of pH in saliva. When using commercial indicators, undesirable leaching might constitute an issue to be addressed. As such, the concentration of the pH indicators used in the devices (i.e., max concentration of ≈1 µg mm^−2^ of coated area) has levels expected to be below thresholds of toxicity in order to make them safer for use. Additional strategies can be adopted including the addition of a semipermeable biocompatible layer that would drive and confine the saliva interaction with the bioactive interface. In the case of the paper points, direct contact of the active interface with the oral cavity can be easily avoided. Due to the high absorbance of the paper, the tip of the point is used to passively draw the saliva into contact with the sensing formulation area that is located farther from the tip.

Another strategy that can be adopted to overcome the drawbacks mentioned above is to rely on naturally available pH indicators such as anthocyanins. These are safe to consume and can be readily extracted from fruits (e.g., blackberries, cherries, blueberries, etc.) and vegetables (e.g., red radishes, red cabbage, red beets, etc.).^[^ ^19^ ^]^

For this purpose, extraction and testing of indicators based on palatable fruits (i.e., BB) and vegetables (i.e., RC) was performed. **Figure** **3**c,d shows the Red channel intensity variation for BB and a combination of BB/RC, respectively, within the pH range 3–8.5. BB and BB/RC points are sensitive within multiple ranges (see the Supporting Information for additional details). Figure 3a,b,e,f shows colorimetric shifts of functionalized paper points at different pH highlighting variations recorded at pH 6 for both BB and BB/RC.

**Figure 3:**
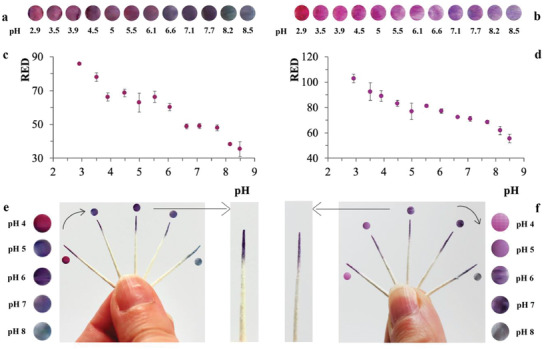
Characterization of biomaterial‐based sensing mixes used to dip coat highly absorbent paper points able to colorimetrically detect pH variations within the oral cavity. The plots and pictures show the color changes at pH values ranging between 3 and 8.5. a,c) Anthocyanins extracted from blueberries (BB) embedded into a silk‐based mix. Colored circles show the colorimetric response recorded at different pH (indicated by the label below circular areas). c) The plot shows sensing ranges of pH 3–4, pH 5.5–6.5, and pH 7.5–8.5 (*n* = 3, error bars standard errors). b,d) Anthocyanins from BB and red cabbage (RC) (i.e., combined in ratio 1:2 v/v) embedded into a silk‐based mix. Colored circles show the colorimetric response recorded at different pH (indicated by the label below circular areas). d) The plot shows sensing ranges of pH 3–5 and pH 7.5–8.5 (*n* = 3, error bars standard errors). e) Highly absorbent paper points dip coated with BB anthocyanins embedded into silk‐based mixes, exposed to different pH 4, 5, 6, 7, and 8 (i.e., the arrow indicates the direction of paper points exposed to increased pH). Left: Colored circles show the colorimetric response recorded at different pH (indicated by the label next to the circular areas). Right: Enlargement of BB point exposed to pH 6. f) Highly absorbent paper points dip coated with BB/RC embedded into silk‐based mixes, exposed to different pH 4, 5, 6, 7, and 8 (i.e., the arrow indicates the direction of paper points exposed to increased pH). Left: Enlargement of RC/BB point exposed to pH 6. Right: Colored circles show the colorimetric response recorded at different pH (indicated by the label next to the circular areas). The plots allow quantifying the signal as variations in the Red channel intensity (i.e., for both types of paper points) versus pH.

Both BB and BB/RC are sensitive over the critical pH range of relevance where they can easily detect changes occurring at pH 5.5, indicative of increased risk for the onset of caries and a critical level at which enamel undergoes demineralization. This strategy would constitute an important and practical in home diagnostic tool that would allow identification of imbalance between cycles of enamel demineralization and remineralization processes without the need of dental X‐rays. Identification of demineralization in such early stages can be utilized to shift the balance into proper remineralization with an adequate supply of calcium, phosphate, and fluoride ions thus providing a method to control the demineralization process and prevent impairment of deeper regions in the enamel layer, where dental caries become irreversible.^[^ ^10^ ^]^

Enamel demineralization of occlusal surfaces can be easily detected by visual examination. On the contrary, enamel demineralization occurring between teeth can be only detected by X‐rays, converting interteeth cavities into harmful players affecting the overall health of the mouth. The use of the functionalized paper points here proposed has considerable utility offering a viable, low‐cost alternative that can be also employed outside the clinical practice for early diagnostic of hard‐to‐detect caries and thus intervene promptly to revert demineralization lesions and avoid dentist intervention.^[^ ^3^, ^21^ ^]^ This method also has the potential to reduce the number of routine X‐rays taken for diagnostics and monitoring purposes.

The use of fruit‐extracted pH indicators allows for alternate approaches for monitoring overall pH variations in saliva that take advantage of the benign nature of the formulation and enables to redefine sensing formats conveniently for everyday use. To demonstrate the utility of this approach, fully edible colorimetric devices in the format of lollipops were realized (**Figure** **4**a,b). These devices embed the naturally available pH indicators into silk fibroin formulations (see the Supporting Information for additional details). Figure 4b shows a lollipop embedding anthocyanins extracted from BB that can be exposed to saliva after introduction inside the oral cavity. The insets show the colorimetric responses recorded at pH 4 and pH 8.5. It is possible to detect variations in the range pH 4–6 (i.e., sensitivity GREEN: −16.3 ± 0.6, *n* = 3), which covers the relevant pH ranges of interest (Figure 4d) to screen for the salivary buffering capacity and overall oral pH levels. pH sensitive consumable devices with different flavors can be realized changing the type of edible indicator. Figure 4c shows the response of bioresponsive lollipops based on carotenoids (i.e., nectarine skin) able to detect pH variations in strongly acidic (i.e., pH 3–3.5, sensitivity GREEN: 70.6 ± 6, *n* = 3), mildly acidic (i.e., pH 4–5.5, sensitivity GREEN: 34.3 ± 4, *n* = 3), and neutral conditions (i.e., pH 6–7, sensitivity GREEN: 34.8 ± 5, *n* = 3). Colorimetric candies can be useful in different scenarios where pH variations in whole saliva need to be continuously monitored or in younger demographics (see the Supporting Information for additional details on chemical composition and degradation over time). The assessment of children's salivary pH is of fundamental importance when kids are undergoing therapeutic drug intakes^[^ ^22^ ^]^ that can affect baseline mouth conditions. From a broader medical perspective, acidic environments are usually observed in the oral cavity of cancer patients^[^ ^23^ ^]^ due to the anaerobic metabolism of glucose in hypoxic conditions created by the tumor acting as a favorable factor for the tumor cells to survive and grow in uncontrolled conditions. Moreover, radiation therapy impairs the function of the salivary glands, which further reduces the oral pH.^[^ ^24^ ^]^ Monitoring the evolution of patients’ salivary pH experiencing cancer treatment^[^ ^23^ ^]^ accounts for the success of radiotherapy that usually causes a shift in pH (i.e., from initial pretty acidic salivary pH to increased pH values during cancer pathogenesis and radiotherapy sessions). This underscores the utility of functionalizing oral devices such as dental aligners/retainers to be used for prolonged periods of time to allow long term monitoring (Figure 4a).

**Figure 4:**
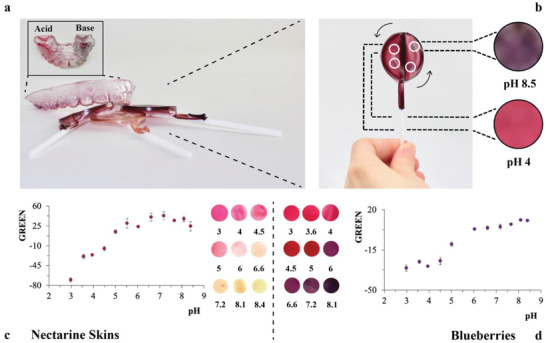
Characterization of mouth‐conformable interfaces based on biomaterial‐based sensing mixes able to colorimetrically detect pH variations within the oral cavity. The plots and pictures show the color changes at pH values ranging between 3 and 8.5. a) Examples of colorimetric mouth‐conformable sensing interfaces that can monitor pH variations within the oral cavity. They can be realized in the format of spray coated dental aligners and edible lollipops able to evaluate the buffering capacity of saliva inside the mouth. The inset shows a dental aligner exposed to pH 3 (i.e., acid) and pH 8.5 (i.e., base). b) Lollipop based on silk fibroin embedding anthocyanins extracted from BB. The lollipop is exposed to pH variations in four highlighted sections: pH 4, 6, 7, and 8.5 (i.e., arrows point at the direction of increased pH). The enlargement shows the color changes recorded at pH 8.5 (i.e., top) and pH 4 (i.e., bottom). c) pH indicator extracted from nectarine skins (N) embedded into a silk mix molded and casted in the format of a lollipop: sensing range pH 3–3.5, pH 4–5.5, and pH 6–7 (*n* = 3, error bars standard errors). Colored circles show the colorimetric response recorded at different pH indicated by the label below every circular area. d) pH indicator extracted from blueberries (BB) embedded into a silk mix molded and casted in the format of a lollipop: sensing range pH 4–6 (*n* = 3, error bars standard errors). Colored circles show the colorimetric response recorded at different pH indicated by the label below every circular area. The plots allow quantifying the signal as variations in the Green channel intensity versus pH for both lollipops.

pH is just the first of many analytes that can be detected by devices similar to those proposed here. All naturally available pH indicators proved to be effective in monitoring variations of biological samples within specific ranges and with preserved stability over time ensured by integration in functional silk fibroin formulations. Other pH indicators are available for extraction from fruits, vegetables, plants, and flowers paving the way for the implementation of palatable pH sensing devices.^[^ ^19^ ^]^

Functionalized mouth‐conformable interfaces that can noninvasively monitor saliva outside the clinical practice offer new possible paradigms to change the management of oral cavity treatment. In the near future, a library of assays may be integrated in dental floss, candies, and dental aligners. The devices will be able to account in a colorimetric fashion for chemical and physical variations occurring in the mouth. Moreover, multibiomarkers detection will provide analytical reports establishing eventual correlations that can account for users’ health conditions.

## Experimental Section

##### Silk Fibroin Solution Preparation

Silk fibroin was prepared as previously reported.^[^ ^25^ ^]^ Briefly, finely chopped *Bombyx mori* silk cocoons were boiled in a solution of 0.02 m sodium carbonate to remove sericin for 120 min. Overnight‐dried silk fibroin was added to 9.3 m LiBr solution and stored at 60 °C to dissolve fibers into aqueous solution. Pure silk solution (≈7–8%) was collected after dialysis (Fisherbrand, MWCO 3.5 kDa) for 48 h.

##### Responsive pH Sensing Cocktails Preparation and Functionalization of Dental Floss

Spray coating of biomaterial‐based sensing cocktails realized mixing pure silk solution (i.e., final concentration of 8%) with commercially available pH indicators (i.e., NY 0.75 mg mL^−1^; BG (0.5 mg mL^−1^)/CPR (0.75 mg mL^−1^)) was carried out with a commercially available airbrush pen spray gun. The cocktail was directly spray coated on both sides of the dental floss sticked in a controlled position to an acrylic holder. Two layers were sprayed on each side of the dental floss that was allowed to dry at room temperature for at least 24 h before testing (see the Supporting Information for additional details).

##### Responsive pH Sensing Cocktails Preparation and Functionalization of Paper Points

Biomaterial‐based cocktails were realized by mixing pure silk solution (i.e., final concentration of 6%) with commercially available pH indicators (i.e., NY 0.75 mg mL^−1^; BG (0.5 mg mL^−1^)/CPR (0.75 mg mL^−1^)), anthocyanins extracted from blueberries (i.e., ratio silk/blueberries solutions 1:5 v/v) and red cabbage (i.e., powder extract 10 mg mL^−1^), carotene extracted from nectarine skins (i.e., ratio silk/nectarine skin solutions 1:5 v/v).^[^ ^19b^ ^]^ The different cocktails embed one or a combination of pH indicators (i.e., BG/CPR, BB/RC 1:2 v/v, BB/RC 2:1 v/v, and BB/N 1:2 v/v) that were used to dip coat highly absorbent paper points. Every paper point tip was dipped in a controlled amount of sensing cocktail (i.e., 1 µL per dip coating layer) multiple times (i.e., NY, 3 layers; BG/CPR, 3 layers; BB, 5 layers; RC, 6 layers; BB/RC 1:2, 6 layers; BB/RC 2:1, 6 layers; BB/N 1:2, 5 layers; N, 6 layers), waiting 30 min between additions. The functionalized paper points were allowed to dry at room temperature for at least 24 h before testing.

##### Responsive pH Sensing Cocktails Preparation and Making of Colorimetric Candies

Pure silk solutions (i.e., initial concentration 14%) were mixed with anthocyanins extracted from blueberries (ratio silk/blueberries 1:1 v/v) or carotene extracted from nectarine skins (ratio silk/nectarines 1:2 v/v). The mixes were transferred on molds to dry at room temperature and obtain edible color changing candies (see the Supporting Information for additional details).

##### Statistical Analysis

All measurements were carried in triplicate (*n* = 3) repeats and results were presented showing the mean ± standard error. The employed statistical methods used to evaluate each experiment are detailed in the subsections of the Supporting Information describing every experimental technique, data processing, and analysis employed during the specific test.

## Conflict of Interest

Tufts University has filed a patent based on this work and will seek to commercialize this technology.

## Supporting information

Supporting Information

## Data Availability

The data that support the findings of this study are available from the corresponding author upon reasonable request.
