# Supplementary material for: Functionalized Mouth‐Conformable Interfaces for pH Evaluation of the Oral Cavity
Source: Adv Sci (Weinh). 2021 Mar 18;8(12):2003416. doi: 10.1002/advs.202003416 (PMC8224410; doi:10.1002/advs.202003416)
Supplement: Supplementary file 1 — Supporting Information [file ADVS-8-2003416-s001.pdf]

WILEY-VCH

© 2021 Wiley-VCH GmbH

ADVANCED SCIENCE
Open Access

Supporting Information

for *Adv. Sci.*, DOI: 10.1002/advs.202003416

Functionalized mouth-conformable interfaces for pH evaluation of the oral cavity

Giusy Matzeu[1,2,3], Gili R.S. Naveh[4], Siddhart Agarwal[1], Jeffery A. Roshko[1], Nicholas A. Ostrovsky-Snider[1], Bradley S. Napier[1], and Fiorenzo G. Omenetto[1,2,3,5,6]*

((Supporting Information can be included here using this template))

Copyright WILEY-VCH Verlag GmbH & Co. KGaA, 69469 Weinheim, Germany, 2018.

Supporting Information

Functionalized mouth-conformable interfaces for pH evaluation of the oral cavity

*Giusy Matzeu, Gili R.S. Naveh, Siddhart Agarwal, Jeffery A. Roshko, Nicholas A. Ostrovsky-Snider, Bradley S. Napier, and Fiorenzo G. Omenetto **

Materials

Sodium carbonate, lithium bromide, sodium phosphate dibasic, citric acid, bromocresol green sodium salt (BG), nitrazine yellow (NY), chlorophenol red (CPR), cyanidin-3-glucose, cyanidin-3-galatoside, cyanadin-3-arabinoside, and malvidin-3-glucoside were purchased from Sigma-Aldrich (USA). Ethanol (100%) was purchased from Fisher Scientific. All chemicals were used as received and they followed trace metal standard, when possible. Artificial saliva was purchased from Pickering Laboratories. Blueberries batches (i.e., from Twin River, Berry Fresh, and Valley Fresh Produce) and nectarines were purchased in a local supermarket. Anthocyanins powders extracted from red cabbage were purchased from Latte Powder. Low sugar Golly Lolli's (commercial lollipops) were purchased in a local store. Silk cocoons of Bombix Mori silkworm were purchased from Tajima Shoji (Japan). Deionized water with resistivity of 18.2 MΩ cm was obtained with a Milli-Q reagent-grade water system and used to prepare aqueous solutions. Dental floss from Reach and highly absorbent

paper points from Dentsply Maillefer were employed as substrates for making mouth conformable colorimetric interfaces.

Silk fibroin solution preparation

Silk fibroin was extracted following previously reported protocols.[25] Briefly, finely chopped Bombix Mori silk cocoons were boiled in a solution of 0.02 M sodium carbonate to remove the sericin layer for 120 minutes. The fibers were washed three times for 20 minutes in deionized water and dried overnight. They were dissolved in a solution of lithium bromide (i.e., 9.3 M) at 60°C for 4 hours. A 20 wt% solution was obtained and dialyzed against deionized water for 2 days, changing the deionized water 6 times at regular intervals. The final solution was centrifuged twice at a speed of 9000 rpm, at 4°C, for 20 minutes and then filtered to obtain a 7-8 wt% silk fibroin solution.

Preparation of biomaterial-based cocktails

Blueberry cocktails: Blueberries were weighted, rinsed with water and completely crushed with a blender. The crushed mixture was transferred in a beaker and the blender was washed with deionized water (ratio water/fruit 1:1 w/w) then added to the blueberry mixture in the beaker. The blueberry cocktail was heated up to 85°C and thickened for 40 minutes. The cocktail was cooled down and filtered 3 times until the final mixture was completely clear and ready to be used to functionalize mouth conformable interfaces. *Carotene extracted from nectarine skins*: Nectarines were rinsed and peeled. Nectarine skins were weighted and packed in a beaker with a mix of

deionized water and ethanol (i.e., ratio 2:1 v/v) double the weight of the starting material. Nectarine skins were kept in infusion overnight at room temperature. The skins were removed from the solution that was filtered to obtain a clear solution ready for use to functionalize and make mouth conformable interfaces. *Biomaterial-based cocktails for spray coating*: 20 cm length of dental floss pieces were cut and assembled on an acrylic holder for spray-coating. An airbrush pen spray gun (Stars Tech Precision) was connected to an air compressor (i.e., working at 5 psi) to control the release of the biomaterial-based sensing cocktails realized mixing pure silk solution (i.e., final concentration of 8%) with commercially available pH indicators (i.e., NY 0.75 mg/mL; BG (0.5 mg/mL) /CPR (0.75 mg/mL)). The cocktail was directly spray coated on both sides of the dental floss kept on the holder. Two layers were sprayed on each side of the dental floss (i.e., waiting 30 minutes between spraying; ~ 500 µL for each layer on 20 cm length of dental floss) that was allowed to dry at room temperature for at least 24 hours before testing. The thickness of the coating layer is the one of the dental floss (i.e., range of 0.23±0.05 mm), completely permeated by the colorimetric cocktail. *Biomaterial-based cocktails for dip coating of paper points:* Biomaterial-based cocktails were realized mixing pure silk solution (i.e., final concentration of 6%) with commercially available pH indicators (i.e., NY 0.75 mg/mL; BG (0.5 mg/mL)/CPR (0.75 mg/mL)), anthocyanins extracted from blueberries (i.e., ratio silk/blueberries solutions 1:5 v/v) and red cabbage (i.e., powder extract 10 mg/mL), carotene extracted from nectarine skins (i.e., ratio silk/nectarine skin solutions 1:5 v/v).[19b] The different cocktails embed one or a combination of pH indicators (i.e., BG/CPR, BB/RC 1:2 v/v, BB/RC 2:1 v/v, BB/N 1:2 v/v) that were used to dip coat highly absorbent paper points. Every paper point tip was dipped in a controlled amount of sensing cocktail (i.e., 1 µL per dip coating layer) multiple times (i.e., NY, 3 layers; BG/CPR, 3 layers; BB, 5 layers; RC, 6 layers; BB/RC 1:2, 6 layers; BB/RC 2:1, 6 layers; BB/N 1:2, 5 layers; N, 6 layers), waiting 30 minutes between layer additions. The functionalized paper points were allowed to dry at room temperature for at least 24 hours before testing. The thickness of the coating layers corresponds to the section of the paper points tips that completely absorbs the

colorimetric cocktail (i.e., on average surface area 6.9±0.2 mm^2, volume of the covered section 0.71±0.02 mm^3). *Biomaterial-based cocktails for colorimetric candies making*: Pure silk solutions (i.e., initial concentration 14%) were mixed with anthocyanins extracted from blueberries (ratio silk/blueberries 1:1 v/v) or carotene extracted from nectarine skins (ratio silk/nectarines 1:2 v/v). The cocktails were transferred on round silicone molds to dry at room temperature and obtain edible color changing candies. The candies were stored in dry conditions at room temperature until further testing.

Anthocyanin extraction from blueberries evaluated via HPLC

High Performance Liquid Chromatography (HPLC) analyses were performed with an Agilent Infinity II 1260 system with autosampler, column heater and variable wavelength detector. The samples were analyzed by UV-Vis detected HPLC, separated using an Agilent Bio-SEC 5 column with isocratic elution of the mobile phase (i.e., eluent pH 4: [0.05 M citric acid]/[0.1 M sodium phosphate dibasic], ratio 1.6 v/v; eluent pH 8: [0.05 M citric acid]/[0.1 M sodium phosphate dibasic], ratio 0.033 v/v). Blueberry extracts (i.e., from three different batches harvested by diverse producers) and anthocyanin standards were diluted using 0.1 M sodium phosphate dibasic and 0.05 M citric acid (i.e., different ratios to have pH 4.0 or 8.0, see details above) as mobile phase. All the samples were then filtered through 0.2 µm PVDF syringe filters before undergoing chromatographic separation.

The chromatographs were normalized and the average and standard errors of normalized chromatographs for three different blueberry extracts at pH 4.0 is shown in **Figure S9a**. **Figure S9a** shows a series of peaks eluted between 18 and 25 minutes, with a ratio of absorbances at 525 and 600 nm (525/600) in the range of 4 to 5. **Figure S9a** shows a high degree of correspondence between the samples since the three different varieties of blueberries extracts showed remarkable consistency in their dye profiles. To help establish the identity of these anthocyanins, a series of molecular standards (i.e., cyanidin-3-glucoside, cyanidin-3-galatoside, cyanidin-3-arabinoside and

malvidin-3-glucoside) of anthocyanins known to be present in blueberries[26] were also chromatographically separated under identical conditions. **Figure S9b** shows that the molecular standards produced a series of distinct peaks between 18 and 25 minutes with 525/600 ratios in the range of 9.8 to 11.8 for the cyanidins and 4 to 6 for malvidin at pH 4. The molecular standards elution times were similar to those of the blueberry extracts under the same acidic experimental conditions.

Figure S9c shows the chromatogram of blueberry extracts at pH 8.0: there is a strong uniform peak at 9 minutes with a 525/600 ratio in the range of 0.9 to 1.1. **Figure S9d** shows the chromatograms of molecular standards characterized by a series of double peaks with a minor peak at 9 minutes and a larger peak recorded at 13-15 minutes with 525/600 ratios in the range of: 0.7 to 1.0 and 1.0 for cyanidins, 1.5 and 0.6 for malvidin for their respective peaks. The ratio of the 9 to 12-14 minutes peak increases in the order that samples were prepared, indicating that the dual peaks may be the product of decomposition or aggregation of the glycosylated anthocyanins at high pH. The 9 minutes peak observed for both blueberry extracts and molecular standards would correspond to the decomposed/aggregated anthocyanins. This phenomenon was not observed under acidic conditions. In conclusion, this chromatographic technique achieved separation between differing molecular standards with identical bound sugars as well as identical molecular standard molecules with differing bound sugars. Therefore, the compound peaks seen in the chromatographs of the blueberry extracts likely represent multiple different anthocyanin compounds, each bound to a variety of different sugars. The relative abundance of these compounds was quite consistent between the blueberry varieties that were sampled.

Viscosity of biomaterial-based cocktails

Viscosity measurements of each biomaterial-based cocktail were taken on a Brookfield DV-II+Pro Viscometer (Middleboro, MA) with a cup and cone (cone CPE-40) geometry and electric gap setting.

Five repeat measurements were taken of each sample with a volume of 0.5 mL after reaching a steady-state viscosity and temperature with a setpoint of 25 °C at 100 rpm (i.e., shear rate 750 s^{-1}). All data are compiled in **Figure S10**. They all have similar viscosity that was found to be slightly lower than that of pure regenerated silk fibroin solutions at a concentration of 5-8wt% (i.e., normally 5-15cP).[27]

Analysis of colorimetric pH sensing interfaces

Biomaterial-based cocktails were first characterized in liquid format using the spectrophotometer Synergy HT from BioTex. Spectra were acquired in the range 400-800 nm, at steps of 5 nm. Mouth conformable interfaces in the form of colorimetric dental floss and highly absorbent paper points were analyzed collecting images using an electronic reader (i.e., 8-bit Laser Jet Pro MFP M127fn scanner from HP (USA), 24-bit color depth and resolution of 600 dpi), or a camera (i.e., Canon EOS Rebel T1i) in controlled lighting conditions. Mouth conformable interfaces in the form of colorimetric edible lollipops can be monitored collecting images using a photo camera. Color changing substrates were photographed with a Canon EOS Rebel T1i in controlled lighting conditions. ImageJ allowed quantifying the signal as variations in the Red or Green channel intensities or a combination of RGB channels intensities expressed in terms of Euclidean Distance (ED) (i.e., $\sqrt{RED^2 + GREEN^2 + BLUE^2}$).

Performances of colorimetric cocktails

Silk-based colorimetric cocktails were characterized via UV-VIS spectrophotometry in the range 400-800 nm, at steps of 5 nm. NY silk-based cocktails were sensitive within the range pH 5.5-8 (n=3) (**Figure S1**). BG/CPR silk-based cocktails were sensitive within the range pH 5.5-7.5 (n=3) (**Figure S2**). **Figure S3** shows the behavior of silk fibroin cocktails embedding anthocyanins extracted from three different batches of blueberries. They display same color maps and sensing ranges (i.e., BB: pH 3-4.2 and pH 5.1-7, (n=3)) with standard deviations in the range of (i.e., for the three batches): 0.01-0.12 for absorbance recorded at 525 nm (i.e., pH range 3-7.4); 0.01-0.04 for absorbance recorded at 610 nm (i.e., pH range 3-7.4). **Figure S4** shows the behavior of silk fibroin cocktails embedding anthocyanins from a commercially available lyophilized powder of red cabbage, sensitive within the pH ranges of 3.5-5.6 and 6-7.8 (n=3). Anthocyanins extracted from diverse fruits and vegetables slightly differ in the chemical structure causing variations in the sensing range and colorimetric response of the overall cocktails.[19] BB and RC can be combined together in different ratios to adjust the color maps accounting for different sensing ranges. **Figure S5** and **Figure S6** show the color map and sensing response of BB/RC in volume ratios of 1:2 (i.e., pH 3.1-4.9 and pH 6.4-7.7; n=3) and 2:1 (i.e., pH 3.2-4.8 and pH 5.8-7.5; n=3), respectively. The different combinations highlight the opportunity to finely tune the sensing range depending on the performances of the final application. Carotene extracted from nectarine skins can also track pH variations via color changes in real-time. **Figure S7** shows the color maps for carotene extracted from nectarine skins. Color differences were noticeable but the sensitivity of silk-based nectarine mixtures was pretty low (i.e., absorbance peaks with max intensity of 0.4 ABS) and the sensing range (i.e., pH 3.3-4.8; n=3) was one unit of pH with a high degree of variability between readings (i.e., standard deviations in the range of 0.001-0.1 for absorbance recorded at 515 nm in the pH range 3.3-7.6). Carotene molecules had to be combined with other color sensing molecules such as anthocyanins extracted from blueberries to improve the overall performances and enlarge the sensing range. Performances were slightly improved (**Figure S8**) since the sensitivity and sensing range were both extended (i.e., pH 3.3-5 and 5.4-7.2, n=3) but the results are not as good as those obtained with commercially available pH indicators and anthocyanins employed at higher concentrations. Carotene based mixtures reduced sensitivity and sensing range seemed to be mainly dictated by the low yield obtained using the extraction procedure mentioned in the section "Preparation of biomaterial-based cocktails". Performances may be easily improved changing the extraction procedure or concentrating the final extract to achieve increased carotene concentrations that will augment the sensitivity of the biomaterial-based cocktails.

Sensing performances of colorimetric mouth conformable interfaces

Dental Floss: Spray coated dental floss embed commercial pH indicators such as NY (**Figure 2a**) or a combination of BG/CPR (**Figure 2e**). Dental floss was cut (i.e., 1 cm length) and exposed to different pH levels (i.e., checked with a standard pH meter) by dipping all the cut area in the pH solution (i.e., 10 μL) for 1 minute before taking a photo/reading of the substrate. Spray coated NY dental floss has Sensitivity RED: -33.8±1.5 (n=3), within the range pH 6-8 (**Figure 2a**). Spray coated BG/CPR dental floss has Sensitivity RED: -26.6±1.6 (n=3), within the range pH 3-6 (**Figure 2e**).

Paper points: Highly absorbent paper points were dip coated with commercially available pH indicators or naturally available anthocyanins and carotene. Paper points were exposed to different pH levels by dipping all the sensing area inside an Eppendorf Tip containing 100 μL of pH solution (i.e., checked with a standard pH meter) for 30 seconds before taking a photo/reading of the substrate. The BG/CPR paper points have Sensitivity ED: -18.3±0.4 (n=3), within the range pH 3-5.5 (**Figure 2b**). The NY paper points have Sensitivity ED: -20±0.5 (n=3), within the range pH 3-7.5 (**Figure 2f**). BB paper points were sensitive within multiple ranges: pH 3-4 (Sensitivity Red: -19.4±1.2, (n=3)); pH 5.5-6.6

(Sensitivity Red: -15.7±1.7, (n=3)); pH 7.7-8.5 (Sensitivity Red: -21.6±2.4, (n=3)) (**Figure 3c**). Paper points realized with a combination of BB/RC (i.e., ratio 1:2 v/v) were sensitive within multiple ranges: pH 3-5 (Sensitivity Red: -18.6±1.3, (n=3)); pH 5.5-7.1 (Sensitivity Green: -6.7±1, (n=3)); pH 7.7-8.5 (Sensitivity Green: -21.6±2.2, (n=3)) (**Figure 3d**). The colorimetric paper points are intended to be positioned in between or in proximity of teeth to verify the presence/absence of cavities by monitoring localized pH variations in real time.

Colorimetric candies: Naturally available pH indicators were also embedded within fully edible mixtures that allowed the realization of color changing lollipop devices. Candies were cut (i.e., squares of 3x3 mm) and exposed to different pH levels by dipping all the cut area in the pH solution (i.e., 100 μL, checked with a standard pH meter)) for 5 minutes before taking a photo of the substrate. The candies are brittle before undergoing exposure to the pH solutions and they become flexible when kept in the liquid. Once dry at the end of the exposure to pH solutions, candies are again brittle but maintain the color dictated by the pH of the solution they were exposed to. BB based lollipops were sensitive within the range pH 4-6 (Sensitivity Green: 16.3±0.6, n=3) (**Figure 4d**). Nectarine based lollipops were sensitive within multiple ranges: pH 3-3.5 (Sensitivity Green: 70.6±6); pH 4-5.5 (Sensitivity Green: 34.3±4); pH 6-7 (Sensitivity Green: 34.8±5) (**Figure 4c**). The pK_a of all pH indicators was shifted after embedment on solid substrates. This phenomenon was previously observed elsewhere and it is attributable to the dye being immobilized within a microenvironment that differs from the standard liquid (i.e., silk in this publication) in which the dyes are usually dissolved.[28] The candies in the format of lollipops are intended to be used as substrates that can be licked by the users and allow monitoring potential variations of salivary pH in real-time.

Evaluation of silk conformation in colorimetric candies

FT-IR Spectral data was collected using a Bruker Invenio S Diamond ATR FT-IR Spectrometer.

FT-IR spectra were measured from 4000 to 400 cm^{-1} with a resolution of $4cm^{1}$, collecting 32 spectra per sample. Each sample was measured in five locations, with three separate samples measured for each lollipop type and one sample measured for regenerated silk fibroin (RSF) mimicking the Blueberry (BB) lollipop without anthocyanin extract (Blueberry Lollipop Blank), RSF and ethanol (EtOH) mimicking the Nectarine (N) lollipop without carotene extract (Nectarine Lollipop Blank), and fruit extracts (i.e., anthocyanins for BB and carotene for N). Blank lollipop spectra were analyzed as previously reported with some modifications[29]. The spectral data of both types of lollipops underwent spectral subtraction, correcting for respective fruit extract peaks in the area of interest. Fourier self-deconvolution (FSD) was completed, the spectra were cut in the Amide I region (1715-1595 cm^{-1}) and baseline corrected using 64 baseline points and 1 iteration of the concave

rubberband correction method. For samples of the BB lollipops, a second-derivative approach was taken, and peak picking along the second derivative in the amide I region was conducted at four local minima. Wavenumber values were fixed in the peak fitting process at these four minima, where the Levenberg-Marquadt method was employed iteratively to fit the spectral data in this region while reducing error. The second-derivative approach proved erroneous with pure BB lollipop blank, N lollipop blank, and N lollipop samples, resulting in incorrectly high β-sheet content. Here, curve fitting was applied by manually selecting four peak locations using gaussian-shaped peaks. A Least-Squares method was then iteratively applied to minimize error while also seeking appropriate peak wavenumber values to fit these spectral data. Areas under the peak in the β-sheet range were compared to the rest of the peaks to calculate β-sheet content, and ANOVA one-way testing was performed with Tukey HSD used for pairwise comparisons.

Figure S11a illustrates the quantified β-sheet content centered at the wavelength 1625 cm^{-1}. The BB lollipop appears to demonstrate a noticeable shoulder in this region, whereas the two blank samples and the N lollipop do not clearly demonstrate this behavior. β-sheet content was quantified as being significantly higher ($p<0.01$) for the BB lollipop as compared to the blueberry lollipop blank, as well as compared to the N lollipop (**Figure S11b**). This is likely caused by interaction of RSF with components of the Blueberry fruit extract. Blueberry extract caused RSF to gel at shorter RSF boiling times (e.g., 30 minutes, RSF higher molecular weight content), suggesting that this may be due to β-sheet formation. Ethanol in the Nectarine lollipop blank sample did not appear to increase β-sheet content compared to the blueberry lollipop blank despite pertaining to the ensemble of alcohols employed as agents to increase β-sheet content of dried RSF-based materials.[29]

Evaluation of colorimetric candies performance in artificial saliva over time

Samples of colorimetric candies (i.e., laser cut in the shape of rectangles 9x13 mm) were kept in 2 mL of diluted artificial saliva (i.e., 1 mL artificial saliva and 1 mL of buffering system consisting of 0.1 M citric acid and 0.2 sodium phosphate dibasic) at pH 4 for 16 hours. 200 µL aliquots were sampled at time intervals of 10, 30, 60, 120, and 960 minutes and they were characterized via UV-VIS spectrophotometry in the range 400-800 nm at steps of 5 nm. **Figure S12a** shows absorbance spectra collected over time. The peak at 525 nm accounts for the leaching of anthocyanin embedded in BB colorimetric candies. **Figure S12c** shows that the anthocyanin release from the silk based candies may follow a cumulative profile[30]. The intensity of the peak increases over time following a linear trend for 60 minutes, showing the onset of a plateau in peak intensity after 60 minutes: the peak increased of 7.5% after 120 minutes. The peak intensity recorded after 960 minutes is 4% higher than the peak recorded after 120 minutes accounting for a minimal indicator release. **Figure S12b** shows that the artificial saliva is purple after 960 minutes and the candy sample still maintains the initial shape dictated by the amount of beta sheets in the film. The sample removed from the solution tends to break and is pretty flexible, opposite outcome when compared to dry candies that maintain their brittleness. **Figure S12d** shows absorbance spectra of aliquoted samples harvested from the artificial saliva storing nectarine (N) candies. The spectra are flat accounting from the absence of carotene release as confirmed by the clear solution shown in **Figure S12e** imaged after 960 minutes. Carotene molecules are really insoluble in water[31] and they do not leach out of the encapsulating sensing substrate maintaining colorimetric performances in a solid format. The N candies keep their shape and they become flexible when exposed to liquid environments. In general, depending on the amount and type of edible indicator embedded in the silk-based candies, it will be possible to tune the secondary structure of the regenerated fibroin allowing for better control in release profiles and degradation over time of the candies here proposed.

Colorimetric lollipops were compared to commercially available counterparts. **Figure S13a** shows absorbance spectra collected over time. The peak at 530 nm accounts for the leaching of the natural

coloring agent (i.e., black currant concentrate) embedded in the candies. There is a burst after 10 minutes accounting for the first release of the colorimetric agent. The intensity of the peak then linearly decreases along 60 minutes (**Figure S13c**) to saturate after 120 minutes (**Figure S13c**). **Figure S13b** shows that the artificial saliva is pink after 120 minutes: the candy was visually dissolved after 30 minutes since it was mainly made out of sugar and tapioca. **Figure S13** suggests that sugar candies cannot really be converted into solid interactive devices able to interact with liquid samples. They allow for single points measurements on a reduced time scale. They hamper the encapsulation of molecules that would eventually leach out too fast complicating the implementation of a library of colorimetric assays that may be feasible exploiting the tunability of silk candy devices (e.g., controlling degradation and release, films vs hydrogels, labile molecules preservation etc.).

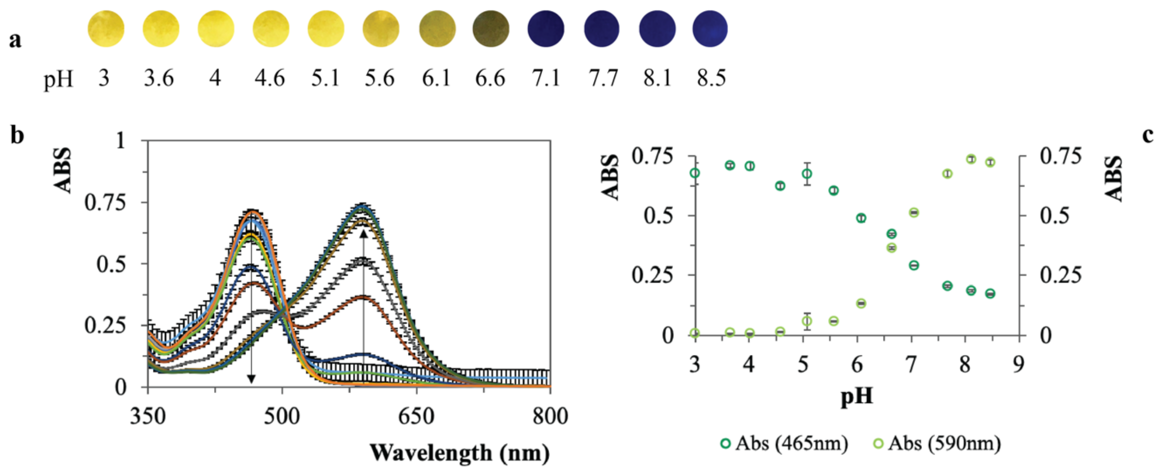

Figure S1. Characterization of NY based pH sensing mix via UV-VIS absorbance measurements. (a) The pictures show the colorimetric response of silk fibroin mixes embedding NY at pH values ranging between 3-8.5 (i.e., label below every circle). (b) Absorbance spectra were recorded within the range 350-800 nm (n=3, error bars standard errors): arrows directions (i.e., in correspondence of two peaks at 465 nm and 590 nm) point at spectra recorded at decreasing or increasing pH values. (c) Intensity variations (i.e., recorded at 465 nm and 590 nm) (n=3, error bars standard errors) are plotted against pH variations (i.e., recorded with a standard pH meter) of each color changing solution.

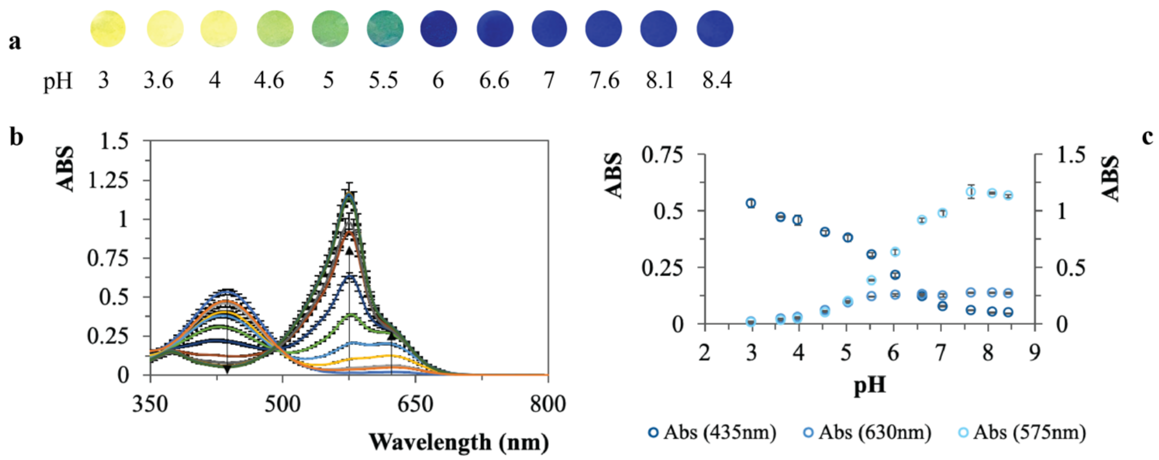

Figure S2. Characterization of CPR/BG (i.e., ratio 1:1) based pH sensing mix via UV-VIS absorbance measurements. (a) The pictures show the colorimetric response of silk fibroin mixes embedding CPR/BG at pH values ranging between 3-8.5 (i.e., label below every circle). (b) Absorbance spectra were recorded within the range 350-800 nm (n=3, error bars standard errors): arrows directions (i.e., in correspondence of three peaks at 435 nm, 575 nm, and 630 nm) point at spectra recorded at decreasing or increasing pH values. (c) Intensity variations (i.e., recorded at 435 nm, 575 nm, and 630 nm) (n=3, error bars standard errors) are plotted against pH variations (i.e., recorded with a standard pH meter) of each color changing solution.

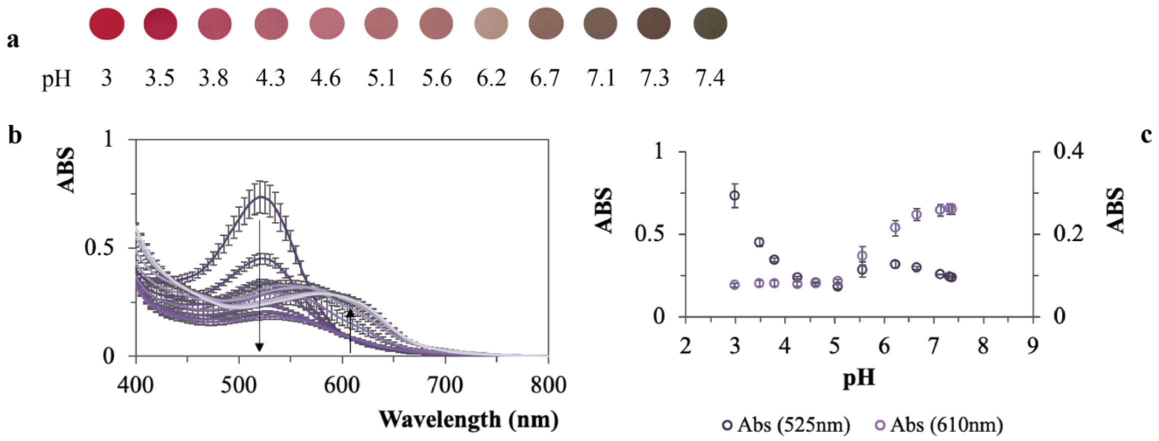

Figure S3. Characterization of BB based pH sensing mix via UV-VIS absorbance measurements. BB were extracted from three different fruit batches harvested in different locations by three different providers. (a) The pictures show the colorimetric response of silk fibroin mixes embedding BB at pH values ranging between 3-8 (i.e., label below every circle). The colorimetric response was the same for the three different BB batches. (b) Normalized absorbance spectra were recorded within the range 400-800 nm (n=3, error bars standard errors): arrows directions (i.e., in correspondence of two peaks at 525 nm and 610 nm) point at spectra recorded at decreasing or increasing pH values. (c) Normalized intensity variations (i.e., recorded at 525 nm and 610 nm) (n=3, error bars standard errors) are plotted against pH variations (i.e., recorded with a standard pH meter) of each color changing solution.

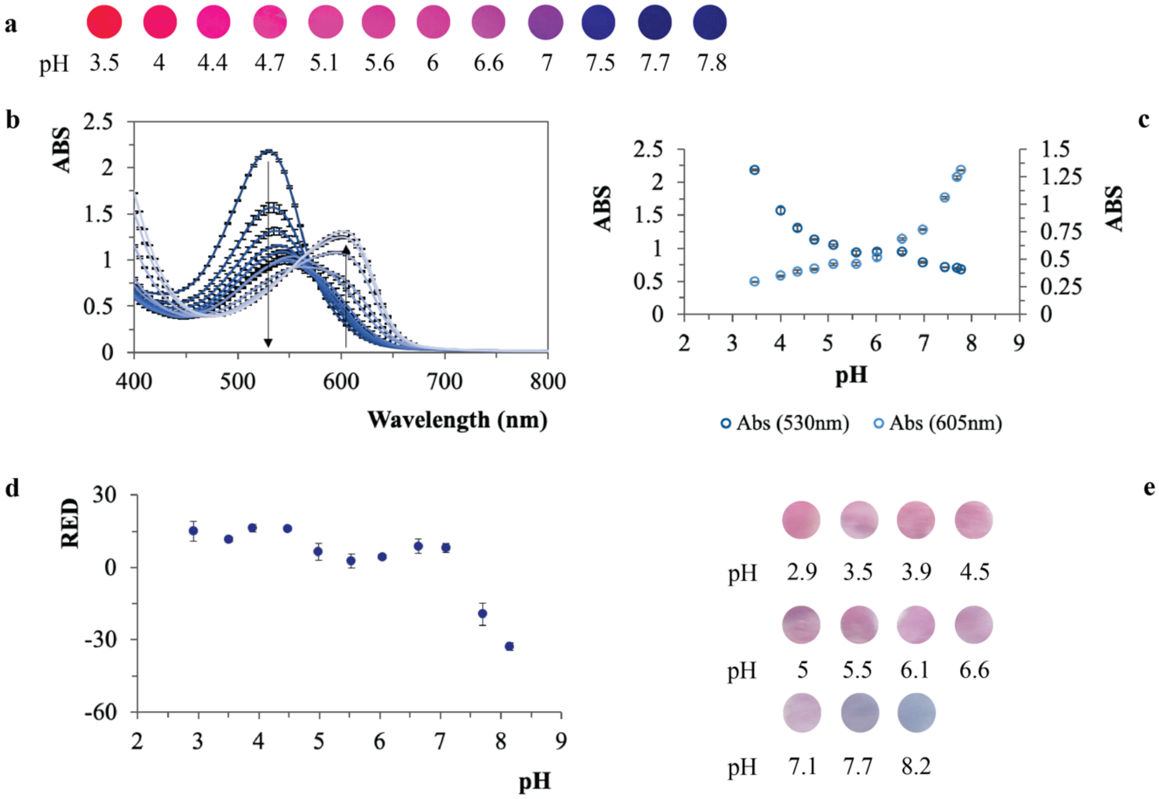

Figure S4. Characterization of RC based pH sensing mix via UV-VIS absorbance measurements. (a) The pictures show the colorimetric response of silk fibroin mixes embedding RC at pH values ranging between 3-8 (i.e., label below every circle). (b) Absorbance spectra were recorded within the range 400-800 nm (n=3, error bars standard errors): arrows directions (i.e., in correspondence of two peaks at 530 nm and 605 nm) point at spectra recorded at decreasing or increasing pH values. (c) Intensity variations (i.e., recorded at 530 nm and 605 nm) (n=3, error bars standard errors) are plotted against pH variations (i.e., recorded with a standard pH meter) of each color changing solution. (d) Sensing response of RC biomaterial-based sensing mixture used to dip coat highly absorbent paper points able to colorimetrically detect pH variations within the oral cavity. The plot shows sensing ranges of pH 4.5-5.5, and pH 7-8 (n=3, error bars standard errors) and it allows quantifying the signal as variation in the Red channel intensity vs pH. (e) Colored circles show the colorimetric response of RC coated paper points recorded at different pH indicated by the label below every circular area.

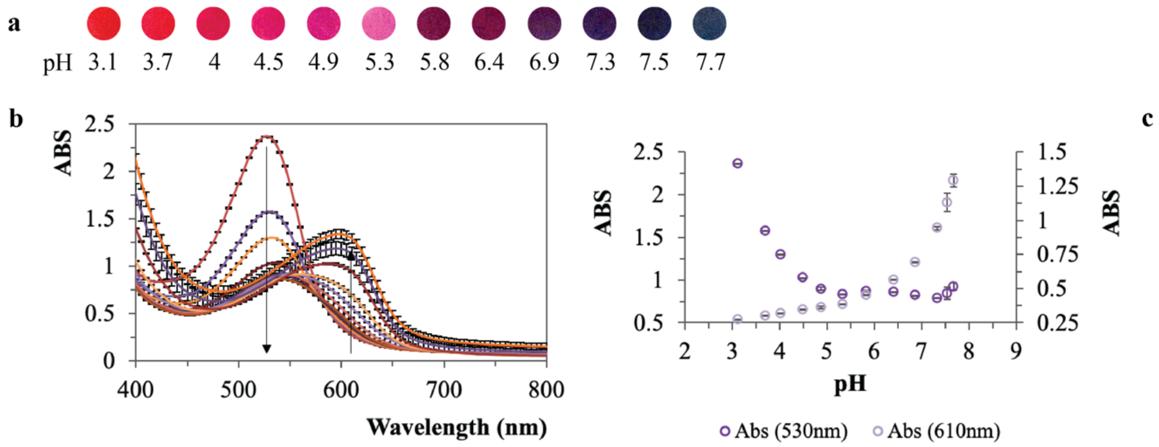

Figure S5. Characterization of BB/RC (i.e., ratio 1:2) based pH sensing mix via UV-VIS absorbance measurements. (a) The pictures show the colorimetric response of silk fibroin mixes embedding RC/BB at pH values ranging between 3-8 (i.e., label below every circle). (b) Absorbance spectra were recorded within the range 400-800 nm (n=3, error bars standard errors): arrows directions (i.e., in correspondence of two peaks at 530 nm and 610 nm) point at spectra recorded at decreasing or increasing pH values. (c) Intensity variations (i.e., recorded at 530 nm and 610 nm) (n=3, error bars standard errors) are plotted against the pH variations (i.e., recorded with a standard pH meter) of each color changing solution.

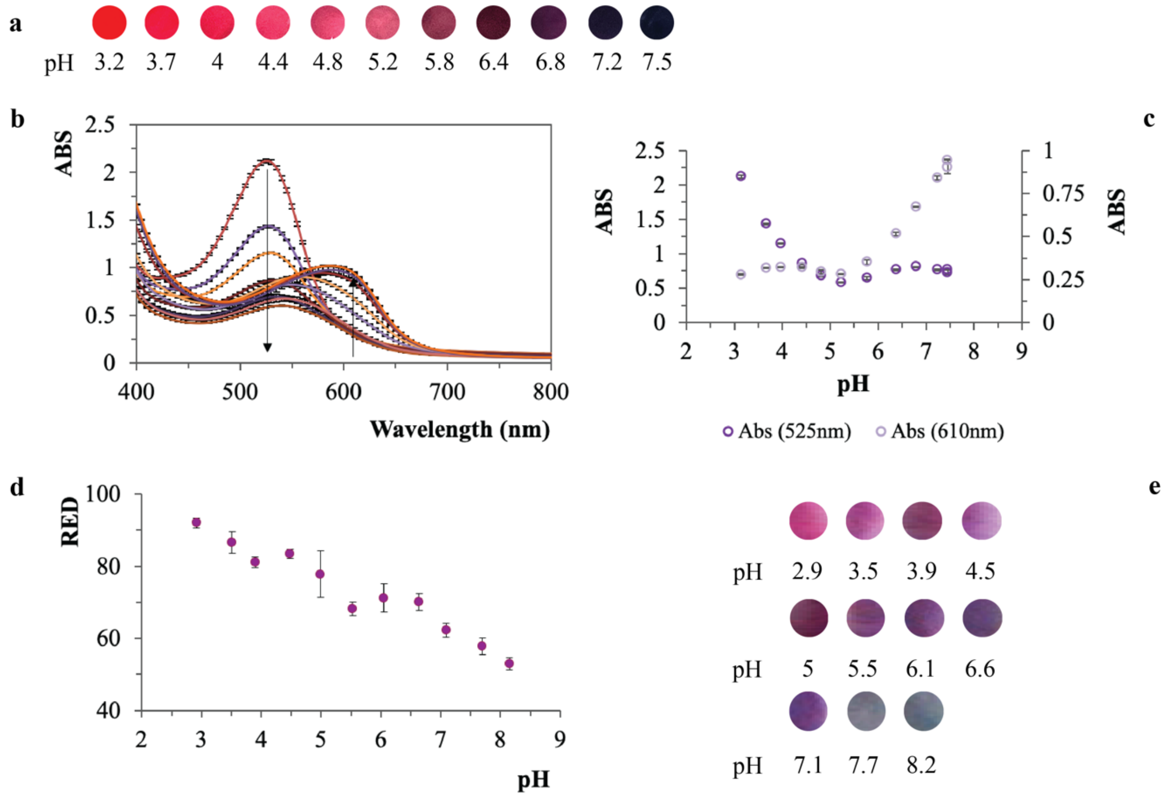

Figure S6. Characterization BB/RC (i.e., ratio 2:1) based pH sensing mix via UV-VIS absorbance measurements. (a) The pictures show colorimetric response of silk fibroin mixes embedding RC/BB at pH values ranging between 3-8 (i.e., label below every circle). (b) Absorbance spectra were recorded within the range 400-800 nm (n=3, error bars standard errors): arrows directions (i.e., in correspondence of two peaks at 525 nm and 610 nm) point at spectra recorded at decreasing or increasing pH values. (c) Intensity variations (i.e., recorded at 525 nm and 610 nm) (n=3, error bars standard errors) are plotted against pH variations (i.e., recorded with a standard pH meter) of each color changing solution. (d) Sensing response of BB/RC biomaterial-based sensing mixture used to dip coat highly absorbent paper points able to colorimetrically detect pH variations within the oral cavity. The plot shows sensing ranges of pH 3-4, pH 4.5-5.5, and pH 6.5-8 (n=3, error bars standard errors) and it allows quantifying the signal as variation in the Red channel intensity vs pH. (e) Colored circles show the colorimetric response of BB/RC coated paper points recorded at different pH indicated by the label below every circular area.

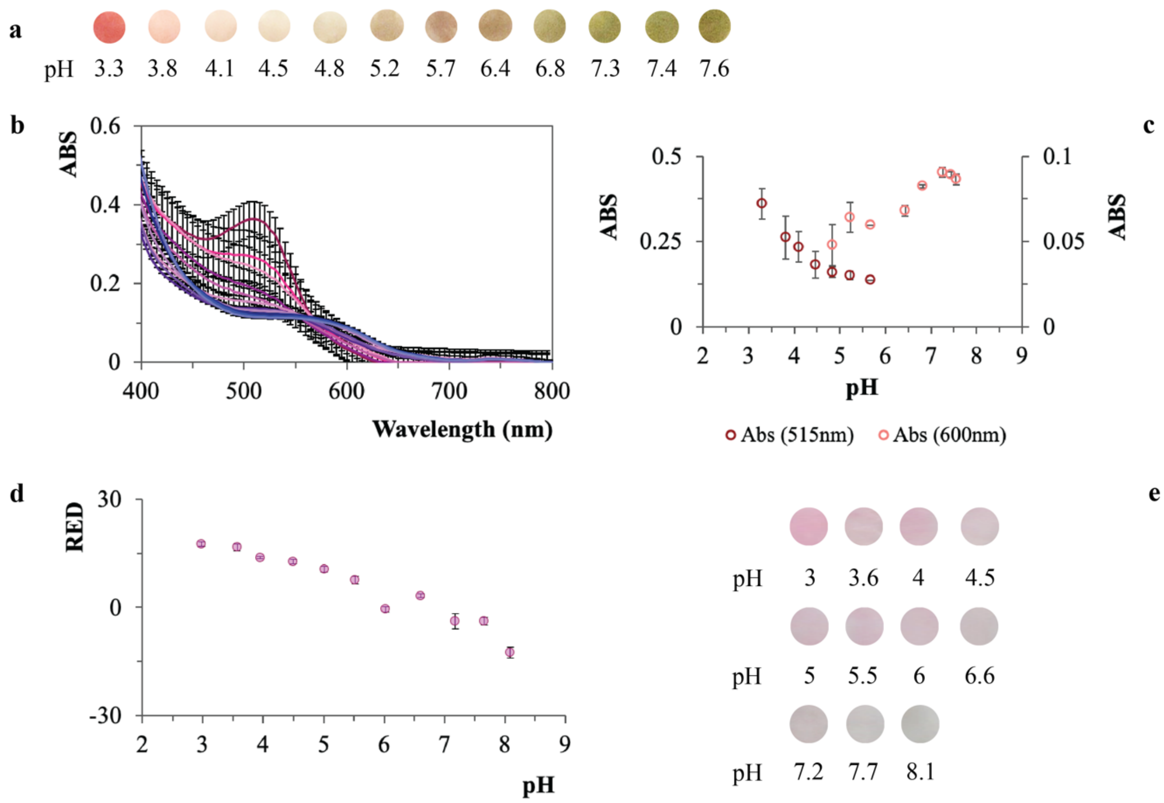

Figure S7. Characterization of nectarine skins (i.e., N) based pH sensing mix via UV-VIS absorbance measurements. (a) The pictures show colorimetric response of silk fibroin mixes embedding N at pH values ranging between 3-8 (i.e., label below every circle). (b) Absorbance spectra were recorded within the range 400-800 nm (n=3, error bars standard errors): arrows directions (i.e., in correspondence of two peaks at 515 nm and 600 nm) point at spectra recorded at decreasing or increasing pH values. (c) Intensity variations (i.e., recorded at 515 nm and 600 nm) (n=3, error bars standard errors) are plotted against pH variations (i.e., recorded with a standard pH meter) of each color changing solution. (d) Sensing response of N biomaterial-based sensing mixture used to dip coat highly absorbent paper points able to colorimetrically detect pH variations within the oral cavity. The plot shows sensing ranges of pH 5.5-6, pH 6.5-7, and pH 7.5-8 (n=3, error bars standard errors) and it allows quantifying the signal as variation in the Red channel intensity vs pH. (e) Colored circles show the colorimetric response of N coated paper points recorded at different pH indicated by the label below every circular area.

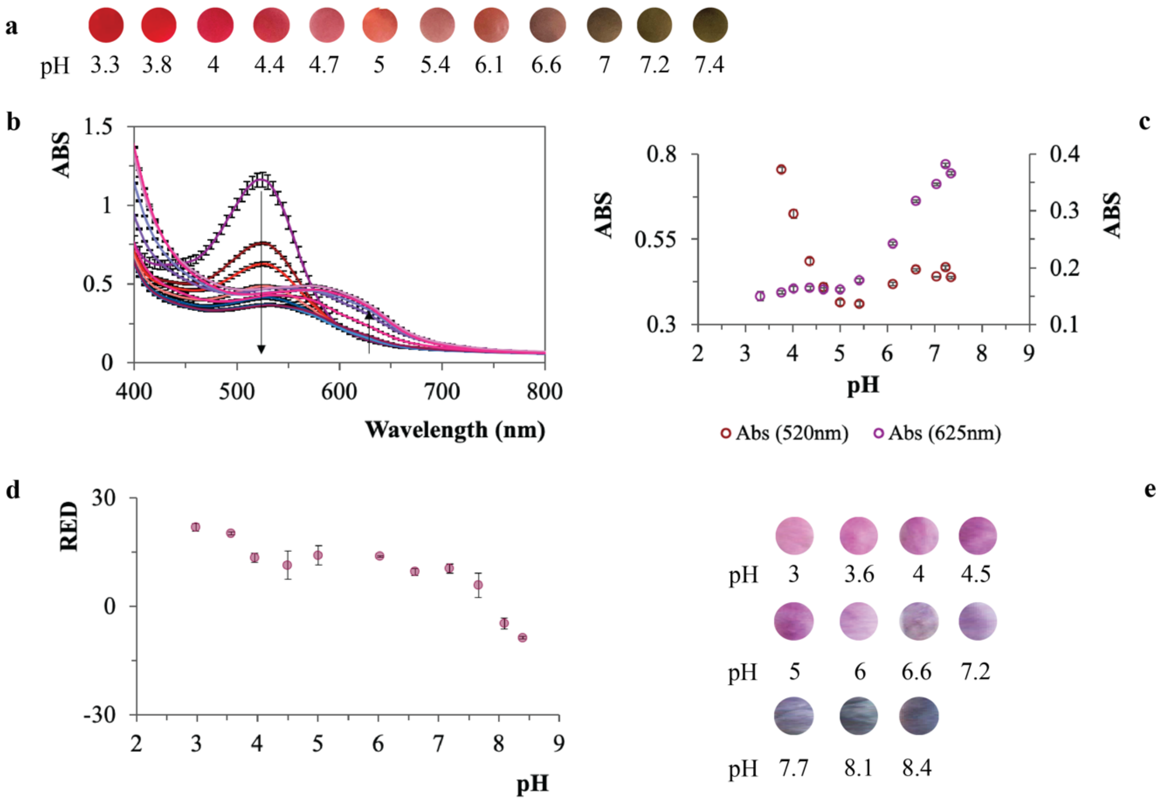

Figure S8. Characterization of N/BB (i.e., ratio 2:1) based pH sensing mix via UV-VIS absorbance measurements. (a) The pictures show colorimetric response of silk fibroin mixes embedding N/BB at pH values ranging between 3-8. (b) Absorbance spectra were recorded within the range 400-800 nm (n=3, error bars standard errors): arrows directions (i.e., in correspondence of two peaks at 520 nm and 625 nm) point at spectra recorded at decreasing or increasing pH values. (c) Intensity variations (i.e., recorded at 520 nm and 625 nm) (n=3, error bars standard errors) are plotted against the pH variations (i.e., recorded with a standard pH meter) of each color changing solution. (d) Sensing response of N/BB biomaterial-based sensing mixture used to dip coat highly absorbent paper points able to colorimetrically detect pH variations within the oral cavity. The plot shows sensing ranges of pH 3.5-4, pH 6.5-7, and pH 7-8.5 (n=3, error bars standard errors) and it allows quantifying the signal as variation in the Red channel intensity vs pH. (e) Colored circles show the colorimetric response of N/BB coated paper points recorded at different pH indicated by the label below every circular area.

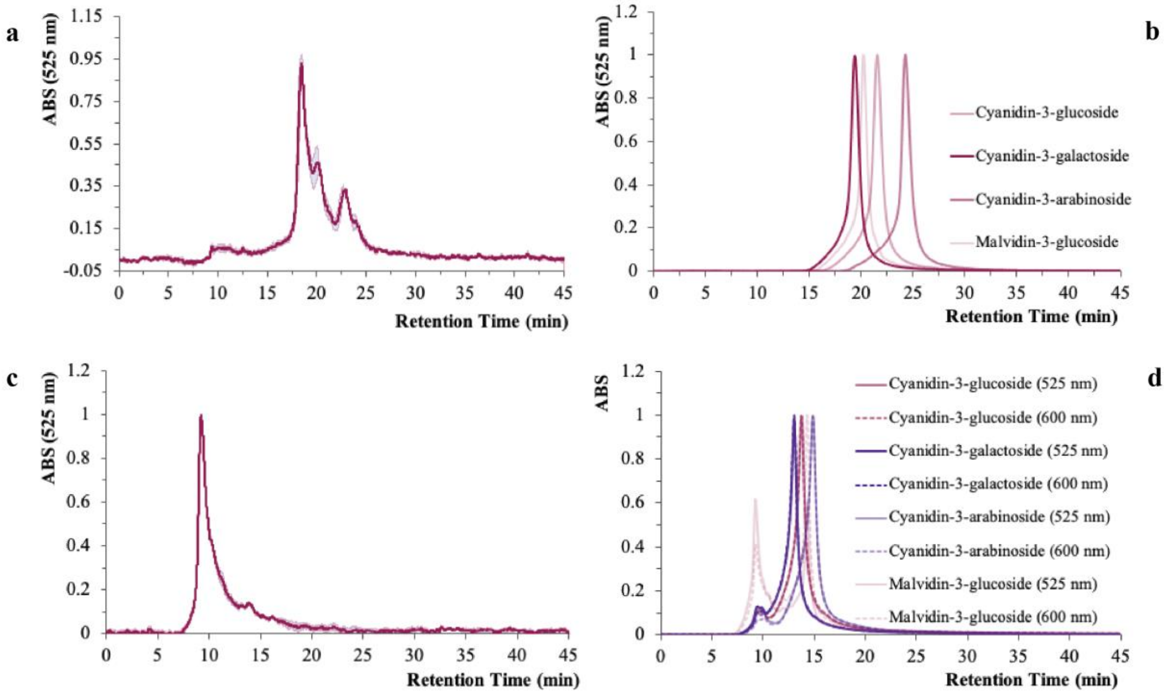

Figure S9. (a) Average normalized chromatographs of three blueberry extracts at pH 4.0 showing the mean (i.e., solid line) and standard error (i.e., shaded region) (n=3). (b) Normalized chromatographs of anthocyanin molecular standards at pH 4.0, detected at 525 nm. (c) Average normalized chromatographs of three blueberry extracts at pH 8.0 showing the mean (i.e., solid line) and standard error (i.e., shaded region) (n=3). (d) Normalized chromatographs of anthocyanin analytical standards at pH 8.0, detected at 525 nm and 600 nm.

Coatings	Viscosity (cP)
Dental Floss:	
BG and CPR	2.7±0.08 (n=5)
NY	2.6±0.1 (n=5)
Paper points:	
Blueberries	3.6±0.1 (n=5)
Red Cabbage	2.7±0.1 (n=5)
Blueberries and Nectarine Skins	2.8±0.05 (n=5)
Nectarine Skins	2.2±0.05 (n=5)
BG and CPR	2.7±0.08 (n=5)
NY	2.6±0.1 (n=5)
Candies:	
Blueberries	3.1±0.1 (n=5)
Nectarine Skins	3.1±0.06 (n=5)

Figure S10. Ink viscosities (i.e., Viscosity, cP) recorded with a cup and cone (cone CPE-40) geometry and electric gap setting (n=5, mean ± standard error).

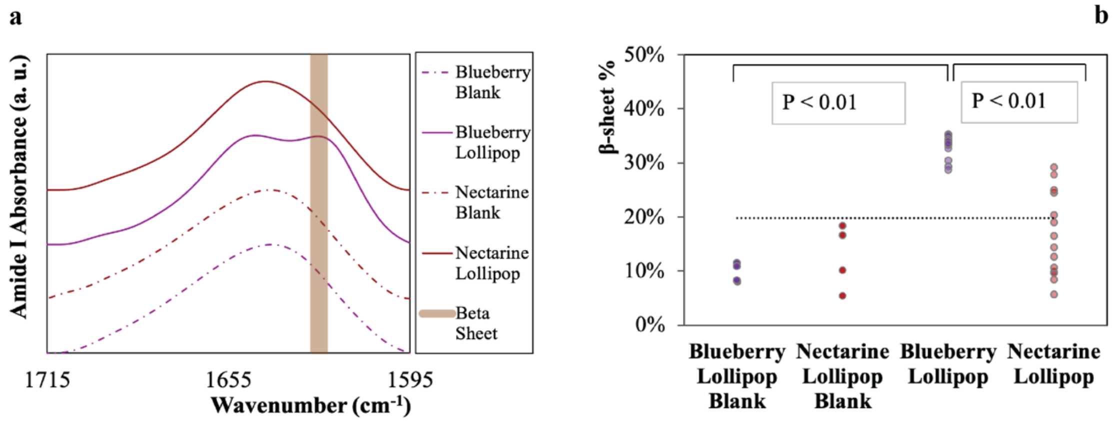

Figure S11. FT-IR and statistical analysis used to quantify regenerated silk fibroin β-sheet content in blueberry lollipop, nectarine lollipop and lollipop blanks (i.e., blueberry and nectarine). (a) FT-IR spectra of the Amide I Region (i.e., 1595-1715 cm^{-1}, FTD). The highlighted area is centered at the wavelength 1625 cm^{-1} to evaluate the presence/absence of the peak corresponding to β-sheet content. (b) ANOVA one-way testing used to calculate the amount of β-sheet in blueberry lollipops, nectarine lollipops, blueberry lollipop blank, and nectarine lollipop blank.

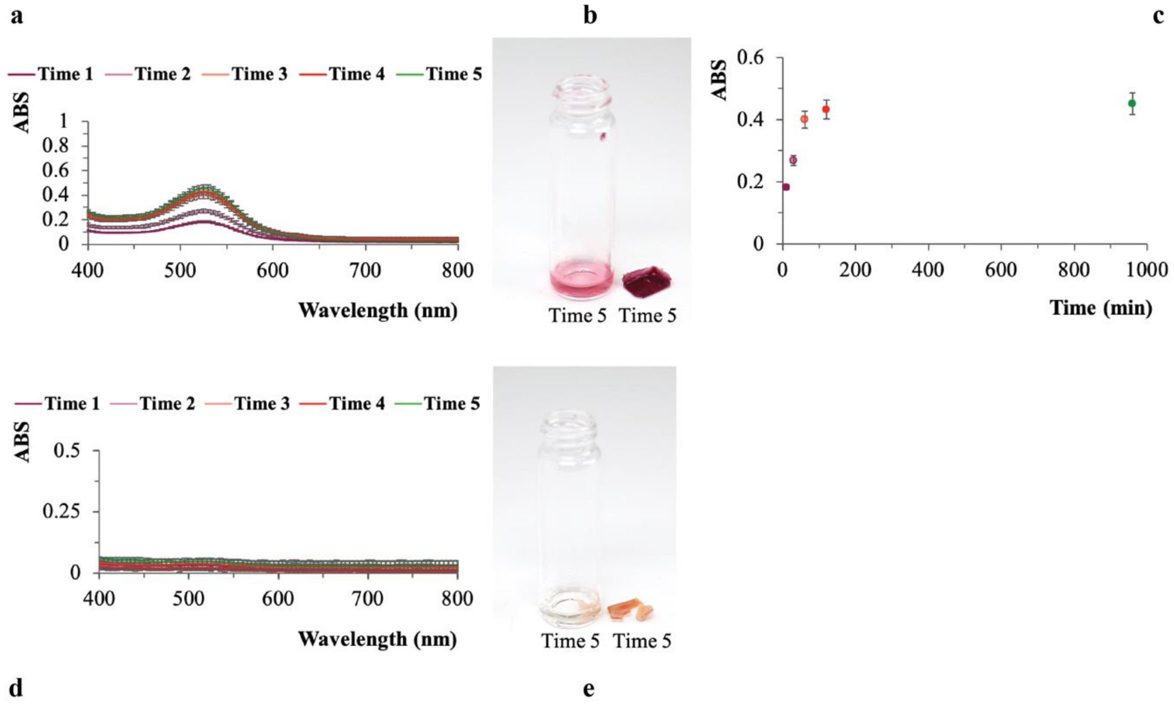

Figure S12. Over time performances of colorimetric candies: samples were kept in artificial saliva at pH 4. (a) Artificial saliva was used to store blueberry (BB) candies. Absorbance spectra of artificial saliva aliquots were recorded over time: Time 1, after 10 minutes; Time 2, after 30 minutes; Time 3, after 60 minutes; Time 4, after 120 minutes; Time 5, after 960 minutes. Spectra were measured within the range 400-800 nm (n=3). (b) The picture shows the purple storage solution (i.e., due to anthocyanin leaching) and a BB candy sample removed from the aforementioned solution (i.e., artificial saliva) after 960 minutes. (c) Intensity variations of sampled aliquots (i.e., recorded at 525 nm, n=3) are plotted against time along 960 minutes. (d) Artificial saliva was used to store nectarine (N) candies. Absorbance spectra of artificial saliva aliquots were recorded over time: Time 1, after 10 minutes; Time 2, after 30 minutes; Time 3, after 60 minutes; Time 4, after 120 minutes; Time 5, after 960 minutes. Spectra were measured within the range 400-800 nm (n=3). (e) The picture shows the clear storage solution (i.e., due to the lack of carotene leaching) and a N candy sample removed from the aforementioned solution (i.e., artificial saliva) after 960 minutes.

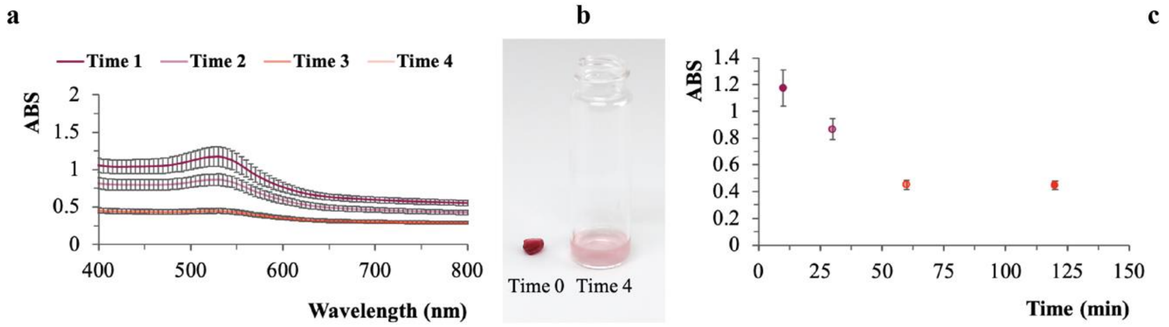

Figure S13. Over time performances of commercially available candies: samples were kept in artificial saliva at pH 4. (a) Artificial saliva was used to store the candies. Absorbance spectra of artificial saliva aliquots were recorded over time: Time 1, after 10 minutes; Time 2, after 30 minutes; Time 3, after 60 minutes; Time 4, after 120 minutes. Spectra were measured within the range 400-800 nm (n=3). (b) The picture shows the pink storage solution (i.e., due to candies dissolution) after 120 minutes. Candies are dissolved after 30 minutes and the Time 0 label is below a sample showing the appearance of candies before exposure to artificial saliva solutions. (c) Intensity variations of every sampled aliquot (i.e., recorded at 530 nm, n=3) are plotted against time along 120 minutes.
